# Fungicidal Activity of a Safe 1,3,4-Oxadiazole Derivative Against *Candida albicans*

**DOI:** 10.3390/pathogens10030314

**Published:** 2021-03-07

**Authors:** Daniella Renata Faria, Raquel Cabral Melo, Glaucia Sayuri Arita, Karina Mayumi Sakita, Franciele Abigail Vilugron Rodrigues-Vendramini, Isis Regina Grenier Capoci, Tania Cristina Alexandrino Becker, Patrícia de Souza Bonfim-Mendonça, Maria Sueli Soares Felipe, Terezinha Inez Estivalet Svidzinski, Erika Seki Kioshima

**Affiliations:** 1 Laboratory of Medical Mycology, Department of Clinical Analysis and Biomedicine, State University of Maringá (UEM), Maringá, Paraná 87020-900, Brazil; renata.daniella@gmail.com (D.R.F.); raquelcmelo97@gmail.com (R.C.M.); glauciasayuria@gmail.com (G.S.A.); karina.msakita@gmail.com (K.M.S.); francieleavr@gmail.com (F.A.V.R.-V.); isiscapoci@gmail.com (I.R.G.C.); patbonfim.09@gmail.com (P.d.S.B.-M.); terezinha.svidzinski@gmail.com (T.I.E.S.); 2 Laboratory of General Pathology, Department of Basic Health Sciences, State University of Maringá, Maringá (UEM), Maringá, Paraná 87020-900, Brazil; tcabecker@uem.br; 3 Program of Genomic Sciences and Biotechnology, Catholic University of Brasilia, Brasília 70790-160, Brazil; msueliunb@gmail.com

**Keywords:** *Candida albicans*, candidiasis, 1,3,4-oxadiazole, drug discovery, antifungal agents, drug resistance, toxicity, biofilm

## Abstract

*Candida albicans* is the most common species isolated from nosocomial bloodstream infections. Due to limited therapeutic arsenal and increase of drug resistance, there is an urgent need for new antifungals. Therefore, the antifungal activity against *C. albicans* and in vivo toxicity of a 1,3,4-oxadiazole compound (LMM6) was evaluated. This compound was selected by in silico approach based on chemical similarity. LMM6 was highly effective against several clinical *C. albicans* isolates, with minimum inhibitory concentration values ranging from 8 to 32 µg/mL. This compound also showed synergic effect with amphotericin B and caspofungin. In addition, quantitative assay showed that LMM6 exhibited a fungicidal profile and a promising anti-biofilm activity, pointing to its therapeutic potential. The evaluation of acute toxicity indicated that LMM6 is safe for preclinical trials. No mortality and no alterations in the investigated parameters were observed. In addition, no substantial alteration was found in Hippocratic screening, biochemical or hematological analyzes. LMM6 (5 mg/kg twice a day) was able to reduce both spleen and kidneys fungal burden and further, promoted the suppresses of inflammatory cytokines, resulting in infection control. These preclinical findings support future application of LMM6 as potential antifungal in the treatment of invasive candidiasis.

## 1. Introduction

*Candida* spp. is the most common cause of nosocomial bloodstream infections by fungal, responsible for over 90% of these cases [1,2]. Immunocompromised patients are the most critically affected, with mortality rates that can reach 60% [3,4]. *C. albicans* remains the most frequent species worldwide, isolated between 20–70% in clinical specimens [5,6]. Among the numerous factors associated with virulence in *C. albicans* which contributes to the high rates of infection and mortality, biofilm is likely to be one of the most important and clinically relevant factors [7]. This fungal organization is able to disrupt host immune response and also the action of antifungal agents on these structures [8]. Therefore, seeking for new and effective treatments against biofilm-associated yeast become necessary.

Currently fungal infections treatment is based on polyenes, azoles or echinocandins [9,10]. Although these agents demonstrate high levels of antifungal activity, their use has serious limitations, in particular due to toxicity, poor tolerability, drug interactions and a narrow activity spectrum [10,11,12]. Moreover, despite the rates are still low, azole and echinocandin resistance has already been reported in isolates of *C. albicans* [13,14]. This scenario has forced the search for alternatives to conventional antimicrobial therapy. The combination of compounds has potential advantages over monotherapy in terms of reducing dose-related toxicity, possibility of action on more than one target and improved antifungal activity [15,16]. Combination therapy may be a solution for antifungal drug resistance.

Another strategy is in silico techniques that have explored virtual screening of chemical libraries against specific targets for drug discovery, reducing time and costs [17,18]. One promising target has been studied is the thioredoxin reductase (Trr1), a flavoenzyme which mainly confers oxidative stress resistance [19]. Recently, the antifungal activity of two compounds of oxadiazoles class (LMM5 and LMM11) which acts on *C. albicans* Trr1 target, were described. These compounds have shown promise against important pathogenic fungi, such as *Candida* spp., *Cryptococcus neoformans* and *Paracoccidioides* spp. with low toxicity in vitro and in vivo [20,21,22,23]. In this study, we evaluated the antifungal activity against *C. albicans* as well as the toxicity in murine model of LMM6 compound, which also belongs to the oxadiazole class and it was selected by in silico approach based on similarity to the LMM11 [20].

## 2. Results

### 2.1. Fungicidal Activity of LMM6

**Figure 1:**
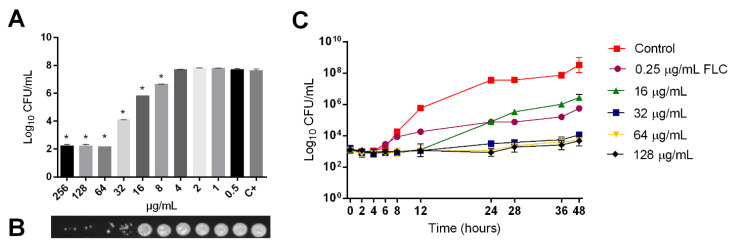
Fungicidal activity of LMM6 against reference strain. (**A**) Quantitative analysis by logarithm reduction of colony forming units. (**B**) Minimum fungicidal concentration (MFC). (**C**) Time-kill curves plotted from log_10_ CFU/mL versus time (0–48 h) for fluconazole conventional drug control (FLC; MIC 0.25 μg/mL) and LMM6 at concentrations 16, 32, 64 and 128 μg/mL, indicating fungicidal profile of new compound. C+ or Control (drug-free control composed medium plus inoculum). Each data point represents the mean ± standard deviation (error bars). * Values of *p* < 0.05 were considered statistically significant.

The susceptibility profile of 30 clinical isolates and reference strains are presented in Table 1. Similar values of MIC for LMM6 (8–32 μg/mL) were reported. All *C. albicans* tested were susceptible to AMB and CAS (100%—31/31). For FLC, 96.8% (30/31) were susceptible and 3.2% (1/31) was resistant. Whereas for ITC, 76.4% (24/31) were susceptible, 19.4% (6/31) dose-dependent-susceptible (SDD) and 3.2% (1/31) was considered as resistant. To confirm the obtained LMM6 antifungal activity, a quantitative analysis was also performed (Figure 1A). The compound effect on CFU was detected at 8–256 μg/mL concentrations with the best activity observed between 64–256 μg/mL in which there was a CFU ≥ 5 log_10_ reduction (*p* < 0.05). MCF results revealed a dose-dependent activity profile for LMM6. The reference strain (Figure 1B) and all clinical isolates (Supplementary Material, Figure S1) showed a notable reduction of fungal growth from 16 and 0.5 μg/mL, respectively, in relation to the positive control. 

LMM6 showed better inhibitory effect than FLC (conventional antifungal) from 6 h in time-kill curve assay (Figure 1C). This activity was sustained for 48 h. Moreover, LMM6 showed fungicidal profile at three concentrations (32, 64 and 128 μg/mL). The reduction in the number of viable cells was ≥ 4 log_10_ (> 99.9%), as compared to control, from 24 h. As expected, the FLC showed a fungistatic profile with CFU reduction ≤ 2 log_10_, as compared to control.

### 2.2. Synergistic Effect between LMM6 and Conventional Antifungals 

**Figure 2:**
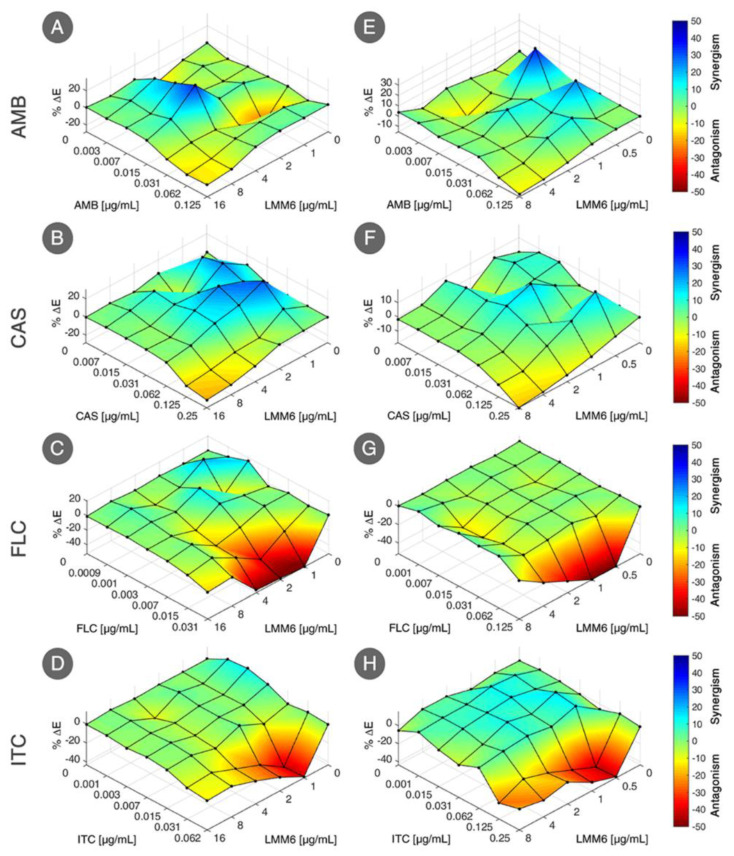
Bliss independence surface analysis for interaction of LMM6 with antifungal conventional drugs (AMB: amphotericin B; CAS: caspofungin; FLC: fluconazole; ITC: itraconazole). Evaluated effect against reference strain (**A**–**D**) and one clinical isolate from hospitalized patient blood (SangHUMCa7; (**E**–**H**)). The x axes represent the antifungal conventional drugs and y axes the LMM6. The magnitude of interactions is directly related to percent ΔE (%ΔE; z axes). Interactions with positive %ΔE represent synergistic effect statistically significant whereas that combinations with negative %ΔE indicate antagonism or no effect. The experimental data were analyzed independently using the Combenefit software.

LMM6 exhibited synergistic interaction with both fungicidal conventional drugs, resulting in a FIC index < 1 for reference strain (AMB: 0.53 and CAS: 0.56) and for one clinical isolate (AMB: 0.75 and CAS: 0.56) (Table 2). However, LMM6 when combined with fungistatic conventional drugs, no synergistic effect was exhibited, resulting in a FIC index > 1 for reference strain (FLC and ITC: 2) and clinical isolate (FLC and ITC: 1.5). The synergic effect of LMM6 with AMB or CAS were confirmed by the presence of blue areas (positive ΔE), in Bliss independence surface analysis (Figure 2A,B,E,F). For the combination of LMM6 with FLC or ITC, red areas were prevalence, which indicates negative ΔE, featuring a without effect combination for these drugs (Figure 2C,D,G,H).

### 2.3. LMM6 Anti-Biofilm Effect

**Figure 3:**
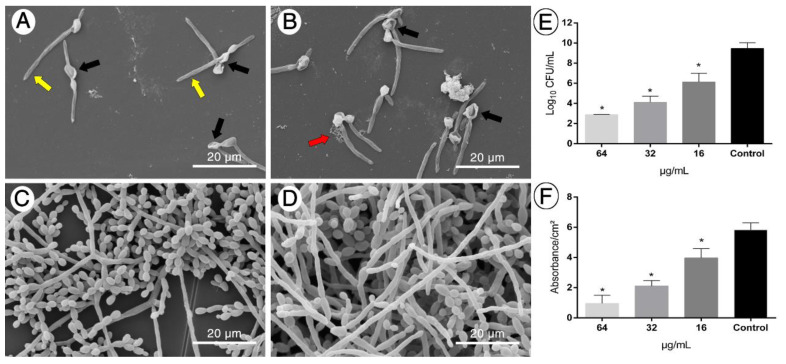
Anti-biofilm effect of LMM6 against reference strain. (**A**–**D**) Scanning electron microscopy (SEM); Representation of the analysis of at least 20 fields. (**E**) Biofilm inhibition by logarithmic reduction of colony forming units (CFU). (**F**) Reduction of total biomass evaluated by staining with crystal violet. LMM6 at concentrations of 64 μg/mL (**A**), 32 μg/mL (**B**) and 16 μg/mL (**C**) were added to the pre-adhered yeast (2h) in polystyrene plate and incubated for 24 h at 37 °C for analysis. Control (**D**): Untreated biofilm containing only RPMI 1640 medium. Black arrows indicate deformities on the cells; Red arrow shown cell extravasation and yellow arrow are membrane and cell wall irregularities. Each data represents the mean ± standard deviation (error bars). * *p* < 0.05, statistically significant reduction in relation to control. The bar in the images corresponds to 20 μm. Magnification × 5000.

Given the clinical relevance of biofilm in invasive fungal infections, the effect of LMM6 on *C. albicans* biofilm structure in formation was investigated. SEM showed that LMM6 was able to disrupt the biofilm growth and cause morphological changes in the cells (Figure 3A–C). In the two highest concentrations of LMM6, 64 μg/mL (Figure 3A) and 32 μg/mL (Figure 3B), the biofilm was characterized by disorganization of structure, presence of deformities in yeast cell, membrane and cell wall irregularities and cell extravasation, when compared to control (Figure 3D). In addition, the reduction in CFU (Figure 3E) was statistically significant (*p* < 0.05) at the three concentrations tested, 16 μg/mL (± 3 log10), 32 μg/mL (± 5 log10), and 64 μg/mL (± 6.5 log10), in relation to untreated control. To check the effect of LMM6 on total biofilm biomass, staining by crystal violet was carried out. All concentrations tested exhibited a statistically significant reduction (*p* < 0.05) of the total biomass (Figure 3F), 16 μg/mL (± 30%), 32 μg/mL (± 60%) and 64 μg/mL (± 80%) compared to the treated control. The morphological, CFU and total biomass alteration was dose dependent.

### 2.4. LMM6 Low Toxicity in Male Balb/c Mice

The acute toxicity in mice was evaluated by intraperitoneal or intravenous LMM6 administration. Single dose of LMM6 caused no death in male mice during 14 days of observation. The reduction of locomotion marked by lethargy and piloerection was observed in all groups treated (LMM6 or vehicle). However, these mild behavioral changes returned to normal after 24 h (Supplementary Material, Table S2). Increased heart rate was also noted in all groups, including healthy mice, and returned to normal soon after manipulation. 

**Figure 4:**
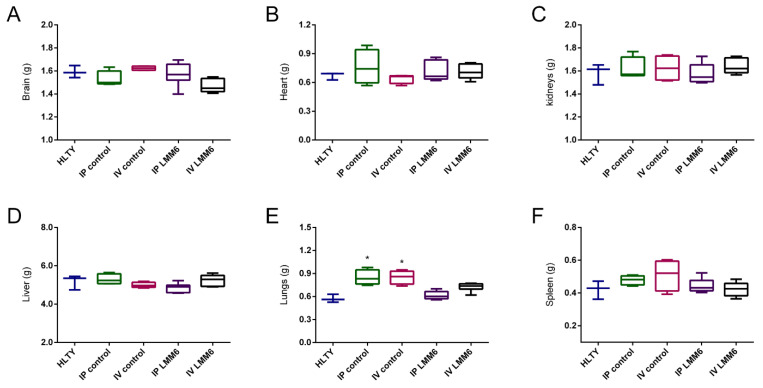
LMM6 effect on organs relative weight of male Balb/c mice in acute toxicity study. (**A**) Brain weight; (**B**) Heart weight; (**C**) Kidneys weight; (**D**) Liver weight; (**E**) Lungs weight and (**F**) spleen weight. HLTY: healthy animals; IP control: treated intraperitoneally with the vehicle; IV control: treated intravenously with the vehicle; IP LMM6: treated with 50 mg/kg of LMM6 intraperitoneally; IV LMM6: treated with 25 mg/kg of LMM6 intravenously. Organs weight were determined following 14 days exposure to high LMM6 concentration in single dose. Each data represents the mean ± standard deviation (error bars). * *p* < 0.05, statistically significant changes in relation to mice.

The relative weight of organs (brain, heart, kidneys, liver, and spleen), subsequent to euthanasia, showed no significant changes after treatment with LMM6 or vehicle (Figure 4). Regardless of the administration route, the vehicle was able to increase the lung weight (*p* < 0.05), as compared to the healthy group (Figure 4E). Macroscopical observations of the organs demonstrated no changes in their color as well as texture. Body weight was not affected by the treatments. No significant difference was recorded when comparing treated mice with healthy (Supplementary Material, Figure S2). 

**Figure 5:**
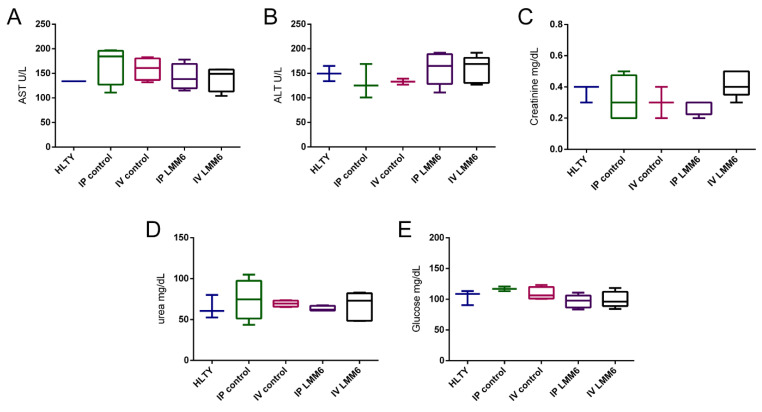
LMM6 effect on biochemical parameters of male Balb/c mice in acute toxicity study. (**A**) Serum levels of aspartate aminotransferase; (**B**) Serum levels of alanine aminotransferase; (**C**) Serum levels of creatinine; (**D**) Serum levels of urea and (**E**) serum levels of glucose. HLTY: healthy animals; IP control: treated intraperitoneally with the vehicle; IV control: treated intravenous with the vehicle; IP LMM6: treated with 50 mg/kg of LMM6 intraperitoneally; IV LMM6: treated with 25 mg/kg of LMM6 intravenous. Biochemical parameters were determined following 14 days exposure to high LMM6 concentration in single dose. Each data represents the mean ± standard deviation (error bars).

The results of biochemical analysis in acute toxicity assay are summarized in Figure 5. Both, LMM6 and vehicle did not cause significant alteration in AST, ALT, CRE, UR and GLU levels when compared to healthy group. Exposure to LMM6 at high concentrations by two administration routes did not lead to liver or renal toxicity. The high dose effect of LMM6 on hematological parameters is presented in Table 3. According to the findings, MCHC index was statistically different (*p* < 0.05) in mice treated (LMM6 or vehicle) when compared to healthy group. The platelet counts only differed for mice treated with vehicle (IP) (*p* < 0.05). Others hematological parameters, as total RBC count, hematocrit, hemoglobin, MCV and MCH were within normal limits and showed no significant change in the analyzed groups. In differential count (Table 4), the leukocytes values increased significantly (*p* < 0.05) in mice treated with vehicle (IV). However, all other counts, neutrophils, monocytes, lymphocytes and eosinophils remained similar among all treated groups with healthy group. Fortunately, this result did not indicate any adverse trend associated with LMM6 treatment and suggests that its application does not promote any abnormalities of blood cells and components in the blood fluids.

### 2.5. Efficacy of LMM6 on the Treatment of Systemic Candidiasis in a Murine Model 

**Figure 6:**
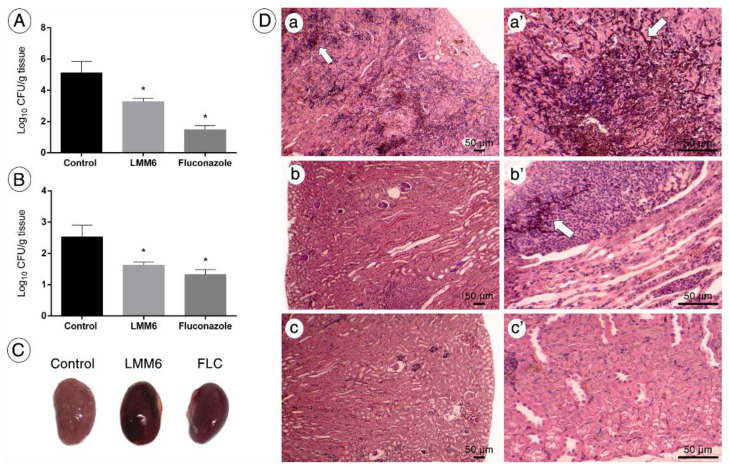
In vivo effect of LMM6 on treatment of mice with systemic candidiasis by *C. albicans*. The mice were infected with reference strain (5 × 10^5^ yeast cells) and treated twice a day for 5 days via intraperitoneal injection. Control: treated with PBS and vehicle (DMSO 1% and Pluronic F-127 0.2%); LMM6: treated with LMM6 compound (5 mg/kg) and FLC: treated with fluconazole (5 mg/kg). (**A**) Fungal burden in the kidneys of the mice. (**B**) Fungal burden in the spleen of the mice. Colony Forming Units (log_10_ CFU) per gram of organ. Each data point represents the mean ± standard deviation (error bars). * Values of *p* < 0.05 were considered statistically significant. (**C**) Surfaces of the kidneys of untreated mice (control) covered with *Candida* lesions, while the kidneys of the LMM6-treated or FLC mice with healthy appearance. (**D**) Kidney histological section stained with hematoxylin eosin and Grocott-Gomori. The bar in the images corresponds to 50 μm. White arrows indicate the presence of fungus in the tissue that were easily spotted in the kidneys of control group (a and a’), whereas few were detected in the kidneys of LMM6-treated mice (b and b’). No fungus was observed on the FLC (c and c’). Representative kidney histopathological sections from 5 mice per group.

The in vivo efficacy of LMM6 was also determined. The groups treated twice a day for 5 days with LMM6 (5 mg/kg) or FLC (5 mg/kg) presented significant reduction in fungal burden in the kidney (CFU; ~1.80 log_10_ and ~3.60 log_10_, respectively) and spleen (CFU; 0.9 log_10_ and ~1.20 log_10_, respectively) when compared to control group (*p* < 0.05; Figure 6A,B). Moreover, visible white lesions covering the kidneys surface were observed in the control group, while in LMM6-treated mice or FLC, the kidneys were apparently healthy (Figure 6C). Histopathological analysis showed tissue damage with increased presence of inflammatory infiltrates and a large amount of yeasts and hyphae in the control group (Figure 6D, a-a’), which decreased considerably in the mice treated with LMM6 (Figure 6D, b-b’). For the FLC group, the fungal cells were not visible in the tissue (Figure 6D, c-c’). 

**Figure 7:**
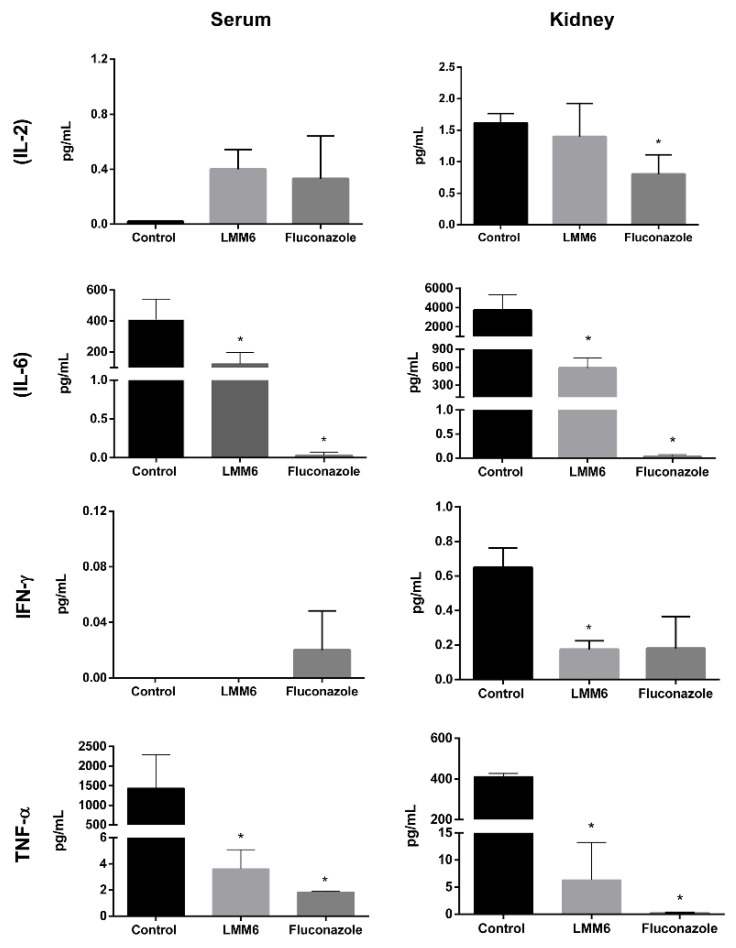
Cytokine detection in the kidney and serum of mice infected systemically with *C. albicans* and treated with LMM6, fluconazole or phosphate buffered saline (PBS). Control: treated with PBS and vehicle (DMSO 1% and Pluronic F-127 0.2%); LMM6: treated with LMM6 compound5 mg/kg; Fluconazole: treated with 5 mg/kg; IL-2: Interleukin-2; IL-6: Interleukin-6; IFN-γ: interferon-γ; TNF-α: tumor necrosis factor-α. The BD^TM^ Cytometric Bead Array (CBA) Mouse Inflammation Kit was used and submitted to BD FACSCalibur™ flow cytometer. Each data point represents the mean ± standard deviation (error bars). * Values of *p* < 0.05 were considered statistically significant in relation to control.

Treatment with LMM6 was able to reduce pro-inflammatory cytokine levels, similar to the action of FLC (Figure 7). Four cytokines (IL2, IL6, IFN-γ and TNF-α) were detected in both serum and kidney. In serum, cytokines IL-2 and IFN-γ showed no significant differences in all groups tested. In the kidneys, IL-2 concentration was reduced for FLC group and IFN-γ decreased for LMM6 (*p* < 0.05). In both serum and kidney, IL-6 and TNF-α presented reduction when treated with LMM6 and FLC as compared to control (*p* < 0.05).

## 3. Discussion

Limited therapeutic arsenal and increase of drug resistance has intensified the search for new antifungals [24]. Our group had sought new therapeutic options by in silico approaches, as virtual screening based on compounds similarity. LMM6 was identified as a direct analogue of a 1,3,4-oxadiazole compound (LMM11), which targets Trr1 from *C. albicans* [17]. This flavoenzyme has been shown an important target for the new drugs development. *TRR1* gene is essential and conserved in several pathogenic fungi. Additionally, it is absent in humans, that can contribute with the development of selective drugs against pathogens [25]. 1,3,4-oxadiazole-based drugs have been widely studied by researchers [21,22,23,26,27,28,29]. These compounds have a high therapeutic power and broad spectrum of action, such as anticancer, antifungal, antibacterial, antitubercular, anti-inflammatory, antineuropathic, antihypertensive, antihistaminic, antiparasitic, antiobesity, antiviral activity, among others [29]. 

In this study, we demonstrated a more promising effect of LMM6, as compared to others 1,3,4-oxadiazoles previously tested in our laboratory [21,22,23]. The LMM6 antifungal activity in vitro clearly reveals reduction in the number of *C. albicans* viable cells (CFU quantitative analysis) in which it was confirmed by growth decrease by CFM. Evidence shows that the fungicidal therapy with echinocandins or amphotericin B yield higher initial cure rates and reduced microbial persistence than fungistatic therapy with triazoles in treatment of invasive *Candida* infection [30]. Our findings indicate that LMM6 presented fungicidal profile with dose and time dependent activity. The fungicidal activity of LMM6 is excellent in comparison with its analogue, which showed fungistatic activity against *C. albicans* [22]. The substitution of the furan-2-yl radical present in LMM11 by 4-fluorophenyl in LMM6 was sufficient to enhance the antifungal effect. Although the MIC90 value was unchanged, the MFC values were reduced from 128 µg/mL (LMM11) to 16ug/mL (LMM6), i.e., the presence of the aromatic ring containing a halogen atom was essential for the fungicidal effect [21,22]. Halogenated compounds are known to show more promising antifungal activity [31]. These results indicate that LMM6 could serve as a scaffold for other substitutions, enhancing the antifungal effects.

The complex biofilm structure contributes to infection persistence, high mortality rates, and is also linked to the enhanced capability of *C. albicans* to resist antifungal [7,8,32]. LMM6 was able to affect the structure of the *C. albicans* biofilm, acting both on yeast reduction and extracellular matrix. These findings were confirmed by SEM, which showed disorganization of architecture, cell extravasation, deformities and irregularities on the yeast cells. In addition, this compound can exert an influence on the membrane surface of the cells preventing adhesion, compromising biofilm formation and leading to surface detachment, as seen in another study [33]. 

The compounds combination is an excellent strategy due to advantages to use lower drug concentrations, wider spectrum of efficacy and reduction of adverse and toxic effects [15,16,34]. This approach has been explored against resistant *C. albicans* strains [35,36,37]. Strong synergistic effects of LMM6 with fungicidal conventional drugs (AMB and CAS) were observed in this investigation. These results represent an attractive prospect for the development of new strategies to manage candidiasis treatment. Differently, the combinations between LMM6 and FLC or ITC have no synergistic effect. The absence of synergistic interaction between fungistatic and fungicidal compounds has been described in the literature. In vitro and in vivo studies have shown that the combination fluconazole and amphotericin B has an indifferent or antagonistic effect on different *Candida* species. [34,38]. 

Acute single-dose toxicity testing using small animal models is imperative in order to predict the adverse effects of a new therapeutic compound. In this study, we demonstrated that both intraperitoneal and intravenous administration of LMM6 were well tolerated by the mice, causing no modifications in clinical status, mortality or general behavior of animals at the tested doses. Changes in the body weights of mice have been used as an indicator of toxic effects of chemicals and drugs [39,40]. Likewise, decreases or increases organs relative weight are also important and sensitive parameter for toxicology studies [41]. Both, body weight and organ relative weight such as brain, heart, kidneys, liver, lungs and spleen were normal indicating no toxic effect in treated groups with LMM6. 

The liver is one of the main target organs for drugs or chemicals due to its important role in the metabolism of these compounds [42]. Elevated serum levels of enzymes such as ALT and AST have been directly linked with liver injury [43]. Kidneys are also frequently sites for drug toxicity because of its function in eliminating xenobiotics and metabolites [44]. Creatinine and urea are commonly used as indicators of renal function [45]. Although not very sensitive and specific, alterations in these parameters may indicate kidney damage [45,46]. We highlight that no significant changes were found in the biochemical analyses between the groups treated with LMM6 and healthy control. Similarly, hematological system analyses may assist in the determination of adverse effects of developing drugs [47]. During the trial, LMM6 did not affect the various hematological parameters evaluated, except for MCHC, which number decreased significantly in all groups. Despite the difference between the groups, the MCHC index remained high in relation to reference standard considered normal for this species [48,49]. 

The in vivo effect of LMM6 treatment seems to be very promising for controlling *C. albicans* infection. Intraperitoneal administration of LMM6 (5 mg/kg, twice a day) was able to reduce both spleen and kidneys fungi burden. Kidneys are one of the main target organs of interest in disseminated candidiasis because besides the other organs do not show persistent colonization by *Candida*, the renal fungal load is directly related to lethality in mice [50,51,52]. Although the results of the fungal burden have been very encouraging, experiments to determine whether LMM6 confers greater animal survival during fungal infection must be performed. In addition, histopathological findings suggest a qualitative reduction of inflammatory infiltrates in mice treated with LMM6 as compared to control. This effect was associated with decrease of fungal load in this organ. Although neutrophils are crucial in the response against fungal pathogens, *C. albicans*-induced excessive infiltration into renal tissue during systemic candidiasis can be deleterious and result in kidney failure and/or mortality [51,53]. 

Pro-inflammatory cytokines are also highly associated with the response of host against *C. albicans* infection [54]. Increased levels of IL-2 suggest that LMM6 contributes to the protection against disseminated candidiasis. In parallel, LMM6 treatment caused reductions in TNF-α and IL-6 cytokines compared to control animals. Suppresses inflammatory cytokines promoted homeostatic restoration in mice similarly to FLC. Similar data were found by Basso et al., in which the treatment efficacy was associated with modulation of pathologic inflammation [55]. These findings can overcome immune response-mediated tissue damage being an advantage in the treatment of *Candida* infections.

## 4. Materials and Methods

### 4.1. Strains and Growth Conditions 

A total of thirty clinical isolates of *C. albicans* from hospitalized patients (collection of fungal culture approved by the Human Research Ethic Committee no. 2.748.843) and one reference strain *C. albicans* ATCC 90028 were used. The clinical isolates were (Supplementary Material, Table S1) identified by classic methods [56] and confirmed with Matrix-assisted laser-desorption/ionization time-of-flight mass spectrometry (MALDI TOF-MS [57]). Prior to each experiment, the strains were cultivated on Sabouraud dextrose agar (SDA; Difco™, MI, USA), and the cellular density was adjusted using a Neubauer chamber. Experiments were performed with the reference strain, except for the antifungal susceptibility and checkerboard assay.

### 4.2. Chemical Compounds

4-[cyclohexyl(ethyl)sulfamoyl]N[5-(4-fluorophenyl)-1,3,4-oxadiazol-2-yl]benzamide (LMM6) was purchased from Life Chemicals Inc. (Burlington, ON, Canada; F2832-0106). Dimethyl sulfoxide (DMSO) was used to prepare LMM6 stock solution (50 μg/mL). Prior to each experiment, LMM6 was solubilized with non-ionic surfactant from Sigma–Aldrich (St Louis, MO, USA; Pluronic^®^ F-127 at 0.02%). Diluents were used as control. The conventional antifungals amphotericin B (AMB) and caspofungin (CAS) were acquired commercially from Sigma–Aldrich (St Louis, MO, USA); itraconazole (ITC) and fluconazole (FLC) were obtained from Pfizer (New York, NY, USA).

### 4.3. Antifungal Susceptibility Testing

The minimum inhibitory concentration (MIC) of LMM6 (0.5–256 μg/mL), AMB (0.032–16 µg/mL), CAS (0.032–16 µg/mL), FLC (0.125–64 µg/mL) and ITC (0.032–16 µg/mL) was determined for 30 clinical isolates and reference strain, based on broth microdilution method, according to document M-27A3 (CLSI) with modifications [58]. The LMM6 and drugs conventional concentrations were prepared in RPMI-1640 medium (Gibco/Invitrogen, Grand Island, NY, USA) and incubated in 96-well plates with yeast (2–3 × 10^3^ cells/mL) for 24 h at 35 °C. Two controls were considered: negative (only medium without inoculum) and positive (medium plus inoculum). The LMM6 MIC values were determined by measuring the absorbance at 405 nm in a SpectraMax ^®^ Plus 384 plate reader (Molecular Devices, Sunnyvale, CA, USA) and defined as the lowest concentration able to inhibit the growth ≥ 80% in relation to positive control. For conventional antifungal agents, MIC values were determined visually: for azoles (FLC and ITC), it was defined as the concentration which resulted in 50% reduction of fungal growth and for polyenes (AMB) and echinocandins (CAS), it was defined as the concentration that was not visible fungal growth in relation to the positive control. The conventional antifungals MICs were interpreted according to the M27-S4 document [59].

The minimum fungicidal concentration (MFC) of LMM6 was determined for all clinical isolates and reference strain by subculture in SDA. The plates were incubated at 35 °C for 24 h. The MFC was defined as the lowest LMM6 concentration at which no colony growth was visible. A quantitative analysis of LMM6 antifungal activity was also performed for each concentration LMM6 tested. After 24 h, aliquots were serially diluted in phosphate buffered saline (PBS), subcultured on SDA medium without the compound and incubated at 35 °C for 24 h for colony-forming units (CFU) counting.

### 4.4. Time-Kill Curve Assay

LMM6 at concentrations of 16, 32, 64 and 128 μg/mL were incubated in 24-well plates at 35 °C with yeast suspension (2–3 × 10^3^ cells/mL) in RPMI-1640. Two controls were prepared: drug-free (only culture medium) and conventional antifungal (FLC; 0.25 μg/mL). At predetermined time points (0, 2, 4, 6, 8, 12, 24, 28, 36, and 48 h), aliquots were obtained from each condition tested, serially diluted in PBS, plated on SDA and incubated at 35 °C for 24 h for CFU determination. Mean counts (log_10_ CFU/milliliter) were plotted as a function of time for each concentration of LMM6 or FLC tested. The fungicidal activity was defined as a ≥ 99.9% (3 log_10_) reduction in numbers of CFU and fungistatic activity was defined as a < 99.9% reduction in growth compared to the control [60,61].

### 4.5. Checkerboard Assay and Bliss-Independent Interactions Analysis

Synergistic interaction between LMM6 and conventional antifungals (AMB, CAS, ITC or FLC) were performed against one clinical isolate (SangHUMCa7 clinical isolate from hospitalized patient blood that has been identified by classical methods and confirmed by MALDI TOF-MS) and reference strain by checkerboard assay [62]. The inhibition of fungal growth was determined by measuring the absorbance at 405 nm on SpectraMax ^®^ Plus 384 plate reader (Molecular Devices, Sunnyvale, CA, USA) after 24 h. The analysis of the combination of the compounds was carried out calculating the fractional inhibitory concentration (FIC) index, which is defined as the sum of FIC^A^ + FIC^B^ (MIC_drug conventional_ in combination/MIC_drug conventional_ alone + MIC_LMM6_ in combination/MIC_LMM6_ alone). Drug interactions were classified as FIC values < 0.5 indicate strongly synergistic effect, FIC < 1 synergistic effect, FIC = 1 additive effect, 1 < FIC < 2 no effect and FIC > 2 antagonistic effect [63]. In addition, all data were analyzed by Combenefit software to obtain Bliss-independent interactions [64]. Interactions with positive %ΔE represent synergistic effect statistically significant.

### 4.6. Effects of LMM6 on Biofilm Formation

The anti-biofilm activity was determined as previously described by Tobaldini-Valerio et al. [65]. A cell suspension prepared in RPMI 1640 medium at a density of 1 × 10^5^ cells/mL was added in 96-well plates (200 μL) and incubated at 37 °C on a shaker at 120 rpm for 2 h to allow adhesion phase. After the incubation, the medium was aspirated from the wells and non-adherent cells were removed by washing with sterile PBS. Subsequently, 200 μL of LMM6 without surfactant F-127, at concentrations 16, 32 and 64 μg/mL were added to form biofilm and incubated at 37 °C/120 rpm/24 h. Untreated biofilm containing only RPMI 1640 medium was used as control. Biofilm inhibition was evaluated by total biomass (crystal violet staining; CV), viability cells (determination of CFU) and scanning electron microscopy (SEM). Before any evaluation, the total medium was removed from wells and the biofilm washed once with PBS to ensure the removal of unadhered cells and the residual of LMM6.

#### 4.6.1. Determination of Total Biomass by Crystal Violet

To assess biofilm total biomass, the wells were fixed with methanol 100% for 15 min. After methanol removal, the 96-well plates were dried at room temperature. CV (0.1% *v*/*v*) was added to the wells (100 μL) for 5 min. The wells were washed twice with sterile distilled water and 200 μL of acetic acid (33% *v*/*v*) was added to dissolve the stain. The absorbance at 570 nm was measured in a SpectraMax ^®^ Plus 384 plate reader (Molecular Devices, Sunnyvale, CA, USA).

#### 4.6.2. Quantification of Viable Biofilm Cells

To determine the number of viable cells, the wells were scraped with PBS using a tip until getting a final volume of 300 μL. The suspensions were vigorously vortexed for 1 min to disaggregate biofilm matrix. Serial dilutions were made in PBS, plated onto SDA and incubated for 24 h at 35 °C for quantification of log_10_ CFU/mL.

#### 4.6.3. Effect of LMM6 on Biofilm Structure

The effect of LMM6 on biofilm structure was evaluated by SEM. The bottom of the 96-well plate was detached, and the biofilm was fixed by immersion in 2.5% glutaraldehyde (Merck KGaA, Darmstadt, HE, Germany) diluted in 2% paraformaldehyde and in 0.1 M sodium cacodylate buffer (Sigma–Aldrich, MO, USA). The samples were dehydrated in an ethanol series (70%, 80%, 90% and 100%) and coated with gold (Baltec SDC 050 sputter coater) for observation using a scanning electron microscope FEI Quanta 250 (Hillsboro, OR, USA) at 5000× magnification [66]. The images are representative of at least 20 fields.

### 4.7. Ethics Statement

All animal procedures were performed according to Brazil’s National Council for the Control of Animal Experimentation (CONCEA) and approved by the Ethics Committee for Animal Use of the State University of Maringá, PR, Brazil (protocol number CEUA 3855010719). The animals were kept with free access to water and food, in a controlled animal facility having a constant temperature of 22–24 °C, relative humidity of 50–60% and a 12 h light/dark cycle.

### 4.8. Evaluation of the Acute Toxicity

The LMM6 acute toxicity in a single dose was evaluated according to the guide for conducting non-clinical toxicology and pharmacological safety studies necessary for drug development (Brazilian Health Surveillance Agency; ANVISA [67]). Inbred male Balb/c mice (n = 23), 6–7 weeks old, weighed 20–30 g, were randomly divided into five groups: HLTY group (healthy animals that just received PBS; n = 3); IP control group (treated intraperitoneally with the vehicle; PBS, DMSO 1%, and Pluronic F-127 0.2%; n = 4); IV control group (treated through the lateral tail vein/intravenous with the vehicle (PBS, DMSO 1%, and Pluronic F-127 0.2%; n = 4); IP LMM6 group (treated with 50 mg/kg of LMM6 intraperitoneally; n = 6) and IV LMM6 group (treated with 25 mg/kg of LMM6 through the lateral tail vein/intravenous; n = 6). The dose administered was calculated according to the body weight for each mice.

The animals were monitored by Hippocratic screening at times 0, 15, 30, 60, 120, 240 min and daily in which were observed clinical and behavioral parameters that included general appearance, motor coordination (touch and tail response, righting reflex and ataxy), muscle tone (paws, body, grip strength), reflexes, lethargy, central nervous systems activity (CNS; tremors, convulsions, muscle contractions, sedation, hypnosis, and anesthesia) and autonomic nervous system (ANS; urination, defecation, piloerection, rate of respiration, heart rate), over a period of 14 days post treatment. Body weight of mice was recorded daily during the whole experiment. After the 14th day, mice were humanely anesthetized for blood collection and then euthanized with isoflurane vaporizer. The organs, brain, heart, kidneys, liver, lungs and spleen were removed, weighed and the relationship between organ weight and body weight (RW) of each mice was established for all groups. Relative weight (RW) = organ weight (g)/body weight on sacrifice day × 100 [68].

#### Biochemical and Hematological Analyzes

Blood collection was done from retro-orbital sinus in microtubes with and without Ethylenediaminetetraacetic acid (EDTA 10%) for hematological and biochemical analysis, respectively. The blood without the EDTA was left at 37° C to coagulate, centrifuged at 5000 rpm, 20 °C for 5 min to obtain serum and stored at −80 °C. Aspartate aminotransferase (AST), alanine aminotransferase (ALT), creatinine (CRE), urea (UR) and glucose (GLU) were determined according to the manufacturer’s instructions using standard diagnostic kits (Gold Analisa Diagnóstica, Brazil) and a semi-automatic biochemistry analyzer BIOPLUS-2000 (Barueri, SP, BR).

Hematology parameters were performed using standard hematological manual methods [69,70]. The analyzes started immediately after blood collection and included: Red blood cells count (RBC; erythrocytes) with hayem liquid, white blood cell count (WBC; leukocytes) with türk solution; platelet count (PLT) by the method of Brecher and Cronkite [71]; hemoglobin concentration (Hb) based on the cyanmethemoglobin method [72] and hematocrit (Ht) by the microhematocrit method. Blood smears were prepared with 20 μL whole blood and May-Grunwald-Giemsa-stained for differential leukocyte count. Using the RBC, Ht and Hb measurement, the hematimetric indexes were calculated as follows: mean corpuscular volume (MCV= Ht × 10/RBC); mean corpuscular hemoglobin (MCH = Hb × 10/RBC) and mean corpuscular hemoglobin concentration (MCHC = Hb/Ht × 100).

### 4.9. LMM6 Antifungal Activity in a Murine Model of Systemic Candidiasis

Systemic candidiasis model was established in female inbred Balb/c mice (n = 18), 6–7-week-old with ± 20 g of weight, according to previously described protocols [22,73]. After injection of 100 µL (5 × 10^5^ yeast cells) of reference strain by the lateral tail vein, the mice were randomly divided into three experimental groups with 6 animals each: LMM6 (treated with LMM6 compound 5 mg/kg), FLC (treated with fluconazole 5 mg/kg) and Control (treated with diluent: PBS buffer, DMSO and Pluronic^®^ F-127). The antifungal treatment started after 3 h of infection and it was conducted twice a day for 5 days via intraperitoneal. The animals were anesthetized for blood collection and then humanely euthanized by isoflurane vaporizer. The left kidney and spleen were aseptically removed for determination of fungal burden. The organs were weighed and then macerated with 1 mL of lysis buffer (200 mM NaCl, 5 mM EDTA, 10 mM Tris, 10% glycerol *v*/*v*, pH 8.30). The homogenates were serially diluted, plated on SDA and incubated for 24 h at 35 °C for CFU counts. Mean CFU counts were normalized by the weight of tissue sample (g).

#### 4.9.1. Cytokines Detection by Flow Cytometry

The kidney homogenates were centrifuged at 14,000 rpm, 4 °C for 15 min and the supernatant transferred to microtubes containing protease inhibitor (GE Healthcare; Chicago, IL, USA). Blood collected from mice retro-orbital sinus was left at 37 °C to coagulate and centrifuged at 5000 rpm, 20 °C for 5 min to obtain serum. The samples were stored at −80 °C prior to analysis. Analysis of the kidney supernatant and blood for detection systemic and local cytokines, respectively, were conducted by BD^TM^ Cytometric Bead Array (CBA) Mouse Inflammation Kit (BD Bioscience, San Jose, CA, USA) as per the manufacturer’s instructions and was analyzed on BD FACSCalibur™ (BD Bioscience, San Jose, CA, USA) flow cytometer. The following cytokines were measured: Interleukin-2 (IL-2), Interleukin-4 (IL-4), Interleukin-6 (IL-6), Interleukin-10 (IL-10), Interleukin-17a (IL-17a), interferon-γ (IFN-γ) and tumor necrosis factor-α (TNF-α). The results for the standard curves of each cytokines and samples were generated using FCAP Array software v3.0 (BD Biosciences, San Jose, CA, USA).

#### 4.9.2. Histopathological Analysis

Immediately after euthanasia, the right kidney of mice was collected and immersed in paraformaldehyde 4% for fixation during 24 h. The organs were preserved in 100% ethanol until processing. Posteriorly, the samples were embedded in paraffin, sectioned longitudinally at 5 μm and stained with hematoxylin eosin (HE) and Grocott-Gomori (GG) for detection of inflammatory areas and fungi in situ. Histopathology images from tissues stained were obtained using a binocular light microscope (Motic BA310- camera Moticam 5), at × 200 and × 600 magnification. In qualitative analysis of fungal cells and inflammatory infiltrates, at least 20 fields of three histological sections were analyzed for each group.

### 4.10. Statistical Analysis

The data were evaluated as the mean ± standard deviation (SD) using Prism 6.0 software (GraphPad, San Diego, CA, USA). Reduction of kidney fungal burden on in vivo treatment was analyzed using unpaired Student’s *t*-test and the other assays with one-way analysis of variance (ANOVA) using the Bonferroni. Values of *p* < 0.05 were considered statistically significant.

## 5. Conclusions

In conclusion, this is the first time the antifungal activity of LMM6 both in vitro and in vivo against *C. albicans* is described. These findings suggest important therapeutic potential of LMM6 due to its fungicidal ability, inhibition of biofilm formation, synergistic effect with conventional drugs and capacity to reduce renal fungal burden in a murine model of disseminated candidiasis. Immunomodulatory activity also increases the protective effects of LMM6 against *C. albicans* infection and maintains homeostasis. LMM6 compound has no hepatotoxic or nephrotoxic effects and does not interfere with the biochemical and hematological parameters in the mice, being safe for future applications as antifungal agent or in association with conventional drugs for the treatment of candidiasis.

## 6. Patents

Kioshima ES, Svidzinski TIE, Bonfim-Mendonça PS, et al. Composição farmacêutica baseada em compostos 1,3,4-oxadiazólicos e seu uso na preparação de medicamentos para tratamento de infecções sistêmicas. Brazil patent BR 10 2018 009020 8. 03 May 2018.

## Figures and Tables

**Table 1:** Antifungal susceptibility of 30 clinical isolates and reference strain to LMM6 and conventional antifungal drugs.

	MIC (µg/mL)			Interpretation † N (%)		
Antifungal Drugs	Range	MIC_50_	MIC_90_	Susceptible	SDD	Resistent
Amphotericin B	0.03–0.5	0.125	0.25	31 (100)	0 (0)	0 (0)
Caspofungin	0.03–0.25	0.125	0.25	31 (100)	0 (0)	0 (0)
Fluconazole	0.06 > 64	0.25	0.25	30 (96.8)	0 (0)	1 (3.2)
Itraconazole	0.03 > 16	0.125	0.25	24 (77.4)	6 (19.4)	1 (3.2)
LMM6	8.0–32	16	32	-	-	-

Abbreviations; MIC: minimal inhibitory concentration; SDD: susceptible-dose-dependent; † MIC interpretation were established by CLSI document M27-S4; MIC_50_ and MIC_90_ were defined as antifungal concentration capable of inhibiting 50% and 90% growth of the isolates, respectively.

**Table 2:** Synergism between LMM6 and conventional antifungals drugs against *C. albicans* by the checkerboard method.

Strains	Combinations	FIC^A^	FIC^B^	FIC Index	Interpretation
Reference strain	AMB/LMM6	0.5	0.031	0.531	Synergistic
	CAS/LMM6	0.5	0.063	0.563	Synergistic
	FLC/LMM6	1	1	2	No effect
	ITC/LMM6	1	1	2	No effect
SangHUMCa7	AMB/LMM6	0.5	0.25	0.75	Synergistic
	CAS/LMM6	0.5	0.063	0.563	Synergistic
	FLC/LMM6	1	0.5	1.5	No effect
	ITC/LMM6	0.5	1	1.5	No effect

Abbreviations; AMB: amphotericin B; CAS: caspofungin; FLC: fluconazole; ITC: itraconazole; FIC index: fractional inhibitory concentration index, calculated as the sum of FIC^A^ plus FIC^B^; FIC^A^: MIC_drug conventional_ in combination/MIC_drug conventional_ alone; FIC^B^: MIC_LMM6_ in combination/MIC_LMM6_ alone. SangHUMCa7 is a clinical isolate from hospitalized patient blood that has been identified by classical methods and confirmed by MALDI TOF-MS.

**Table 3:** Hematological parameters of male Balb/c mice exposed to high LMM6 concentration in acute toxicity assessment.

Hematological Parameters	Analyzed Groups				
	Healthy	IP Control	IV Control	IP LMM6	IV LMM6
Total RBC (10^6^/mm^3^)	8.39 ± 0.53	8.96 ± 0.92	9.13 ± 0.32	8.96 ± 0.58	8.31 ± 1.28
Haematocrit (%)	43.66 ± 0.58	45 ± 1	45.5 ± 1	44.43 ± 0.53	46 ± 0
Hemoglobin (g/dL)	21.48 ± 0.64	20.31 ± 0.40	20.90 ± 0.69	20.65 ± 0.58	21.01 ± 0.21
MCV (fL)	52.15 ± 3.08	50.31 ± 5.11	49.86 ± 0.98	49.77 ± 3.67	53.46 ± 3.40
MCH (pg)	25.64 ± 1.15	22.83 ± 2.06	22.89 ± 0.46	23.05 ± 1.49	24.02 ± 1.44
MCHC (%)	49.21 ± 1.83	44.88 ± 0.50 *	45.92 ± 0.73 *	46.49 ± 1.37 *	45.27 ± 1.03 *
Platelet (10^3^/mm^3^)	355.66 ± 71.28	570.00 ± 125.79 *	406.00 ± 42.68	402.71 ± 48.33	394.00 ± 77.24

Hematological parameters were determined following 14 days exposure to LMM6 in single dose. Abbreviations; Healthy: normal mice; IP control: treated intraperitoneally with the vehicle; IV control: treated intravenous with the vehicle; IP LMM6: treated with 50 mg/kg of LMM6 intraperitoneally; IV LMM6: treated with 25 mg/kg of LMM6 intravenous. RBC: Red blood cells; MCV: mean corpuscular volume; MCH: mean corpuscular hemoglobin; MCHC: mean corpuscular hemoglobin concentration. Values represent the mean ± SD. * *p* < 0.05, statistically significant changes compared with healthy control.

**Table 4:** Differential count of peripheral blood leukocytes of male Balb/c mice exposure to high LMM6 concentration in acute toxicity assessment.

Groups	Leukogram					
	Leukocytes	Neutrophils	Monocytes	Lymphocytes	Eosinophils	Basophils
	10^3^/mm^3^	% (10^3^/mm^3^)				
Healthy	4.5 ± 1.9	18 ± 4.36 (0.83 ± 0.62)	1 ± 1 (0.05 ± 0.05)	78.66 ± 5.14 (3.52 ± 1.47)	2.33 ± 0.58 (0.11 ± 0.06)	-
IP control	7.9 ± 2.0	17.25 ± 1.70 (1.36 ± 0.35)	0.5 ± 1 (0.04 ± 0.09)	80 ± 3.74 (6.37 ± 1.82)	2.25 ± 1.5 (0.16 ± 0.12)	-
IV control	10.0 ± 2.6 *	20 ± 3.91 (2.05 ±0.77)	0.5 ± 0.58 (0.05 ± 0.06)	77.75 ± 4.19 (7.77 ± 2.02)	1.75 ± 1.5 (0.15 ± 0.12)	-
IP LMM6	5.4 ± 2.4	18.14 ± 4.45 (1.06 ± 0.62)	1 ± 0.63 (0.06 ± 0.06)	78.86 ± 4.88 (4.21 ± 1.70)	2.14 ± 1.21 (0.11 ± 0.11)	-
IV LMM6	6.0 ± 1.9	19.66 ± 2.94 (1.14 ± 0.25)	0.66 ± 0.51 (0.05 ± 0.04)	77.66 ± 2.16 (4.71 ± 1.65)	2 ± 0.82 (0.13 ± 0.08)	-

Differential count of peripheral blood leukocytes was realized following 14 days exposure to LMM6 in single dose. Abbreviations; Healthy: normal mice; IP control: treated intraperitoneally with the vehicle; IV control: treated intravenous Differential count of peripheral blood leukocytes were realized following 14 days exposure to LMM6 in single dose. Abbreviations; Healthy: normal mice; IP control: treated intraperitoneally with the vehicle; IV control: treated intravenous with the vehicle; IP LMM6: treated with 50 mg/kg of LMM6 intraperitoneally; IV LMM6: treated with 25 mg/kg of LMM6 intravenous. Values represent the mean ± SD. * *p* < 0.05, statistically significant changes compared with healthy control.

## Data Availability

The data presented in this study are available within the article or supplementary material.

## Supplementary Materials

The following are available online at https://www.mdpi.com/2076-0817/10/3/314/s1. Table S1: Specimen/source of 30 clinical isolates of *C. albicans* and antifungal susceptibility profile to LMM6 and conventional antifungal drugs; Table S2: General appearance and behavioral observations of male Balb/c mice exposed to high LMM6 concentration in the acute toxicity study; Figure S1: Minimum fungicidal concentration of LMM6 for 30 clinical isolates of *C. albicans*. After exposure of yeast to increased LMM6 concentrations (0.25–128 μg/mL) for 24 h, aliquots of 3 μL of the solution were transferred to SDA plates and incubated at 35 °C. Representative photograph of three independent experiments; Figure S2: Comparative body weight changes in male Balb/c mice during acute toxicity experimentation. The body weight of mice was weighed daily for 14 days following single dose administration of LMM6. Healthy: normal mice; IP control: treated intraperitoneally with the vehicle; IV control: treated intravenous with the vehicle; IP LMM6: treated with 50 mg/kg of LMM6 intraperitoneally; IV LMM6: treated with 25 mg/kg of LMM6 intravenous.
