# Supplementary material for: Fungicidal Activity of a Safe 1,3,4-Oxadiazole Derivative Against Candida albicans
Source: Pathogens. 2021 Mar 7;10(3):314. doi: 10.3390/pathogens10030314 (PMC8001722; doi:10.3390/pathogens10030314)
Supplement: Supplementary file 1 [file pathogens-10-00314-s001.zip › Supplementary Material pdf/Supplementary Material Figure S1.pdf]

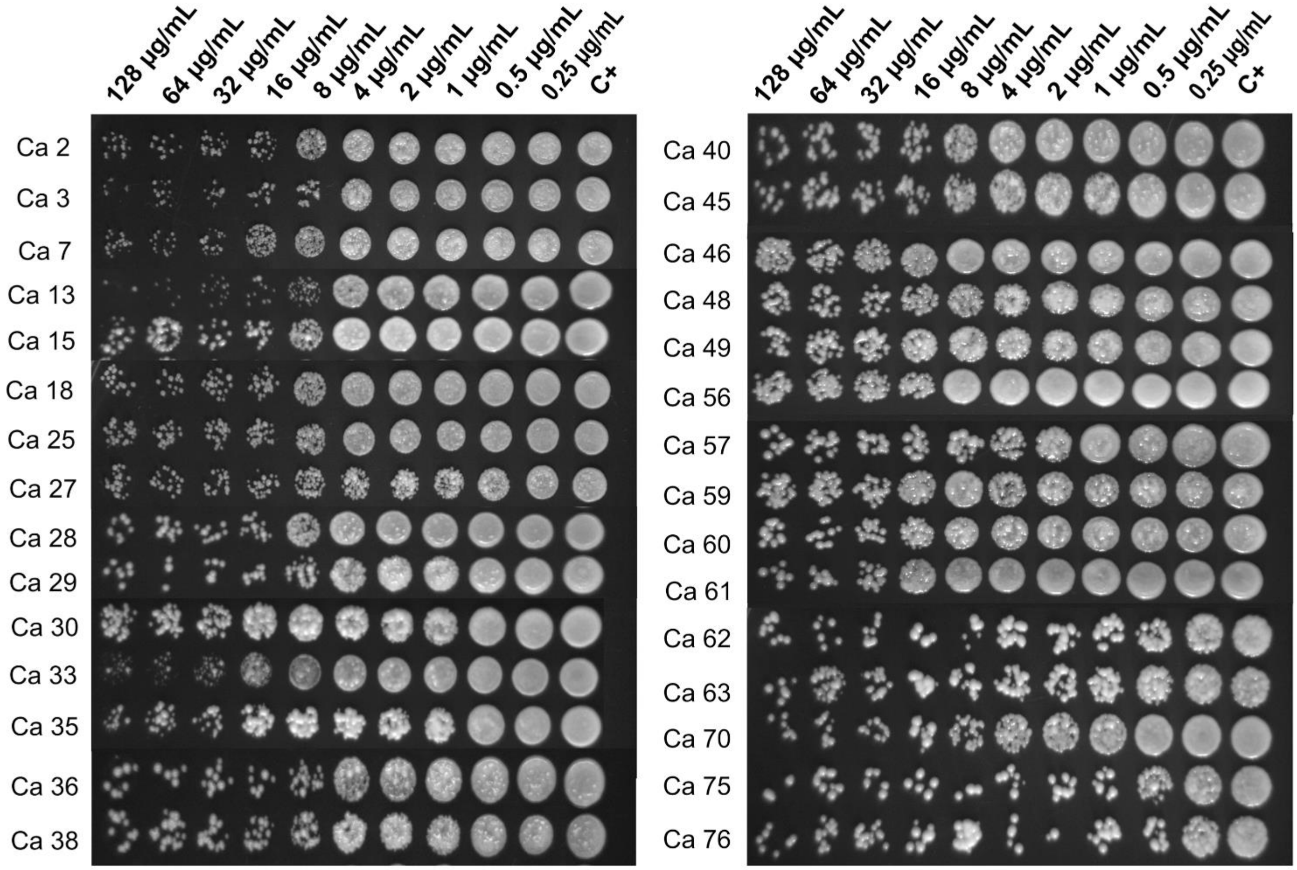

Supplementary Material Figure S1. Minimum fungicidal concentration of LMM6 for 30 clinical isolates of *C. albicans*. After exposure of yeast to increased LMM6 concentrations (0.25-128 µg/mL) for 24 hours, aliquots of 3µL of the solution were transferred to SDA plates and incubated at 35°C. Representative photograph of three independent experiments.
