# Supplementary material for: Fungicidal Activity of a Safe 1,3,4-Oxadiazole Derivative Against Candida albicans
Source: Pathogens. 2021 Mar 7;10(3):314. doi: 10.3390/pathogens10030314 (PMC8001722; doi:10.3390/pathogens10030314)
Supplement: Supplementary file 1 [file pathogens-10-00314-s001.zip › Supplementary Material pdf/Supplementary Material Figure S2.pdf]

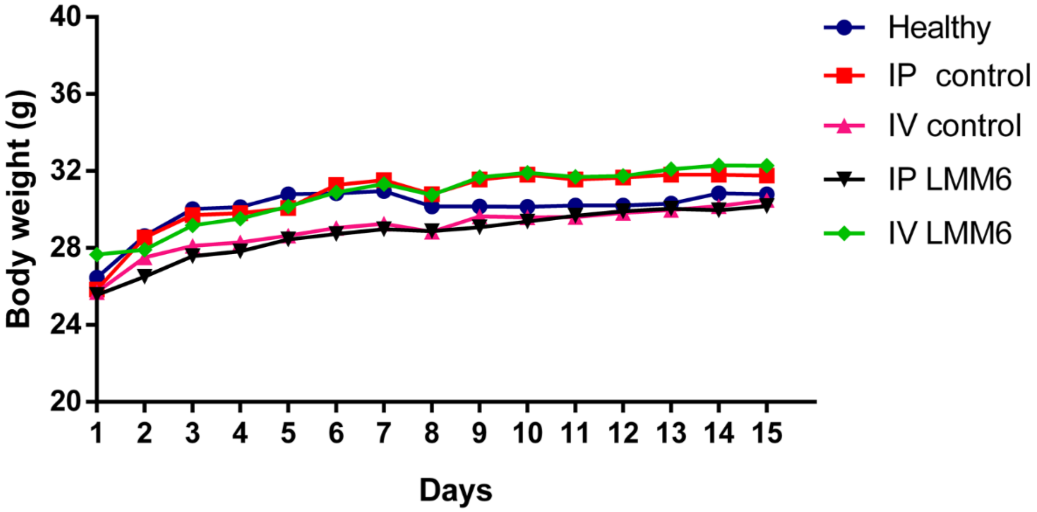

Supplementary Material Figure S2. Comparative body weight changes in male Balb/c mice during acute toxicity experimentation. The body weight of mice was weighed daily for 14 days following single dose administration of LMM6. Healthy: normal mice; IP control: treated intraperitoneally with the vehicle; IV control: treated intravenous with the vehicle; IP LMM6: treated with 50mg/kg of LMM6 intraperitoneally; IV LMM6: treated with 25 mg/kg of LMM6 intravenous.
