# Supplementary material for: Fungicidal Activity of a Safe 1,3,4-Oxadiazole Derivative Against Candida albicans
Source: Pathogens. 2021 Mar 7;10(3):314. doi: 10.3390/pathogens10030314 (PMC8001722; doi:10.3390/pathogens10030314)
Supplement: Supplementary file 1 [file pathogens-10-00314-s001.zip › Supplementary Material pdf/Supplementary Material Table S1.pdf]

Supplementary Material Table S1. Specimen/source of 30 clinical isolates of *C. albicans* and antifungal susceptibility profile to LMM6 and conventional antifungal drugs.

	Clinial isolates	Specimen/ Source	MIC µg/mL				
			Amphoterin B	Caspofugin	Fluconazole	Itraconazole	LMM6
1	**UrHUMCa2**	Urine	0.25	0.125	0.125	0.125	8
2	**UrHUMCa3**	Urine	0.125	0.125	0.06	0.5	8
3	**SangHUMCa7**	Blood	0.25	0.125	0.125	0.25	8
4	**UrHUMCa13**	Urine	0.06	0.03	>64	>16	4
5	**UrHUMCa15**	Urine	0.03	0.25	0.125	0.06	8
6	**UrHUMCa18**	Urine	0.125	0.25	0.125	0.06	16
7	**SangHUMCa25**	Sangue	0.06	0.125	0.125	0.03	8
8	**UrHUMCa27**	Urine	0.125	0.125	0.125	0.06	8
9	**SangHUMCa28**	Sangue	0.03	0.125	0.125	0.06	8
10	**LasHUMCa29**	Ascitic fluid	0.03	0.03	0.125	0.06	8
11	**UrHUMCa30**	Urine	0.5	0.25	0.25	0.25	32
12	**UrHUMCa33**	Urine	0.25	0.25	0.25	0.125	16
13	**SangHUMCa35**	Sangue	0.25	0,25	0,25	0.125	16
14	**LAbHUMCa36**	Abdominal fluid	0.03	0.06	0.125	0.06	8
15	**UrHUMCa38**	Urine	0.06	0.03	0.125	0.06	8
16	**UrHUMCa40**	Urine	0.03	0.125	0.125	0.06	8
17	**UrHUMCa45**	Urine	0.06	0.125	0.25	0.125	8
18	**UrHUMCa46**	Urine	0.125	0.125	0.25	0.06	16
19	**UrHUMCa48**	Urine	0.25	0.125	0.25	0.125	32
20	**UrHUMCa49**	Urine	0.125	0.125	0.125	0.06	16
21	**UrHUMCa56**	Urine	0.125	0.25	0.25	0.125	16
22	**PtHUMCa57**	Catheter tip	0.125	0.06	0.25	0.125	16
23	**UrHUMCa59**	Urine	0.125	0.125	0.25	0.125	16
24	**UrHUMCa60**	Urine	0.25	0.125	0.25	0.06	16
25	**UrHUMCa61**	Urine	0.125	0.125	0.5	0.125	8
26	**PtHUMCa62**	Catheter tip	0.125	0.125	0.5	0.25	32
27	**UrHUMCa63**	Urine	0.125	0.125	0.25	0.06	16
28	**UrHUMCa70**	Urine	0.125	0.125	0.25	0.125	16
29	**LAbHUMC75**	Abdominal fluid	0.25	0.06	0.25	0.25	32
30	**UrHUMCa76**	Urine	0.25	0.06	0.125	0.25	16

Abbreviations; MIC: minimal inhibitory concentration.
