# Supplementary material for: Fungicidal Activity of a Safe 1,3,4-Oxadiazole Derivative Against Candida albicans
Source: Pathogens. 2021 Mar 7;10(3):314. doi: 10.3390/pathogens10030314 (PMC8001722; doi:10.3390/pathogens10030314)
Supplement: Supplementary file 1 [file pathogens-10-00314-s001.zip › Supplementary Material pdf/Supplementary Material Table S2.pdf]

Supplementary Material Table S2. General appearance and behavioral observations of male Balb/c mice exposed to high LMM6 concentration in the acute toxicity study.

Observations	Healthy		IP control		IV control		IP LMM6		IV LMM6	
	0’-24h	2°-14° dia	0’-24h	2°-14° dia	0’-24h	2°-14° dia	0’-24h	2°-14° dia	0’-24h	2°-14° dia
General appearance	N	N	N	N	N	N	N	N	N	N
Motor coordination	N	N	NC	NC	NC	NC	NC	NC	NC	NC
Muscle tone	N	N	NC	NC	NC	NC	NC	NC	NC	NC
Reflexes	N	N	NC	NC	NC	NC	NC	NC	NC	NC
Lethargy	A	A	P	A	P	A	P	A	P	A
Tremors	A	A	A	A	A	A	A	A	A	A
Convulsions	A	A	A	A	A	A	A	A	A	A
Sedation	A	A	A	A	A	A	A	A	A	A
Hypnosis	A	A	A	A	A	A	A	A	A	A
Anesthesia	A	A	A	A	A	A	A	A	A	A
Urination	N	N	NC	NC	NC	NC	NC	NC	NC	NC
Defecation	N	N	NC	NC	NC	NC	NC	NC	NC	NC
Piloerection	A	A	P	A	P	A	P	A	P	A
Rate of respiration	N	N	NC	NC	NC	NC	NC	NC	NC	NC
Heart rate	↑	N	↑	N	↑	N	↑	N	↑	N
Death	A	A	A	A	A	A	A	A	A	A

Behavioral parameters were observed at times 0, 15, 30, 60, 120, 240 minutes and daily following single dose administration of LMM6. Abbreviations; N: normal; NC: Not changed; P: present; A: ausent; ↑ : Increased; Healthy: normal mice; IP control: treated intraperitoneally with the vehicle; IV control: treated intravenous with the vehicle; IP LMM6: treated with 50mg/kg of LMM6 intraperitoneally; IV LMM6: treated with 25 mg/kg of LMM6 intravenous.
